# Objective Non-Invasive Bio-Parametric Evaluation of Regenerated Skin: A Comparison of Two Acellular Dermal Substitutes

**DOI:** 10.3390/life14010121

**Published:** 2024-01-15

**Authors:** Silvia Rampazzo, Marco Ferrari, Maria Alessandra Sotgiu, Gabriella Piu, Maria Giuliana Solinas, Noemi Usai, Antonio Bulla, Pietro Luciano Serra, Federica Grieco, Andrea Montella, Vittorio Mazzarello, Corrado Rubino

**Affiliations:** 1 Plastic Surgery Unit, University Hospital Trust of Sassari, 07100 Sassari, Italy; n.usai7@studenti.uniss.it (N.U.); abulla80@gmail.com (A.B.); p.serra4@studenti.uniss.it (P.L.S.); f.grieco1@studenti.uniss.it (F.G.); corubino@uniss.it (C.R.); 2 Plastic, Reconstructive and Aesthetic Surgery Training Program, University of Sassari, 07100 Sassari, Italy; 3 Skinlab, Department of Biomedical Science, University of Sassari, 07100 Sassari, Italy; dr.marcoferrari@gmail.com (M.F.); gpiu1@uniss.it (G.P.); vmazza@uniss.it (V.M.); 4 Department of Biomedical Science, University of Sassari, 07100 Sassari, Italy; asotgiu@uniss.it (M.A.S.); gsolinas@uniss.it (M.G.S.); montella@uniss.it (A.M.); 5 Department of Medicine, Surgery and Pharmacy, University of Sassari, 07100 Sassari, Italy

**Keywords:** dermal substitutes, skin graft, biophysical properties, regenerated skin, Integra^®^, Pelnac^®^

## Abstract

Several dermal substitutes are available on the market, but there is no precise indication that helps surgeons choose the proper one. Few studies have tried to compare different xenogeneic bioengineered products, but no objective bio-parametric comparison has been made yet. Fifteen patients who underwent skin reconstruction with Integra^®^ or Pelnac^®^ were retrospectively evaluated. After at least 12 months of follow-up, an objective and quantitative assessment of several skin biophysical properties, such as color, texture, elasticity, hydration, glossiness and trans-epidermal water loss, were measured with non-invasive skin measurement devices. The grafted skin showed a reduction of the superficial hydration level and a tendency to lower values of trans-epidermal water loss with both dermal substitutes. Melanic and hemoglobin pigmentation were higher in comparison to the donor site in both groups, while a melanic pigmentation increase versus the surrounding skin was seen just with Integra^®^. Finally, the skin was found to be more elastic when reconstructed with Integra^®^. The skin barrier appeared to be intact in both groups. Hence, these substitutes are valuable means of skin regeneration. Integra^®^ seems to be more advantageous for reconstructing areas that need more skin flexibility.

## 1. Introduction

The skin represents the largest organ in our body and explicates numerous well-known properties. Acting as a barrier, it is involved in immunologic, thermic and metabolic functions [1]. The skin is basically composed of two layers: the superficial epidermis and the deeper dermis. The epidermis is composed of different layers of keratinocytes in various stages of maturation, ranging from the deepest layer consisting of stem cells to the outermost stratum corneum, which forms a physical barrier against water loss and external agents. The epidermis also contains Merkel cells and Langerhans cells with neuroendocrine and immunological functions, respectively. The dermis is composed of epithelial cells immersed in a connective tissue matrix rich in collagen, glycosaminoglycans, elastic fibers, blood vessels, sweat and sebaceous glands and hair follicles [2,3].

Full-thickness defects, resulting from trauma, surgical intervention or chronic wound, consist of a complete loss of both epidermis and dermis tissue and represent a challenge for the reconstructive surgeon. The fundamental treatment for full-thickness defects is skin grafts and flaps [4,5], with the latter representing the gold standard in most cases. Flaps have already been proven to be a reliable option for the reconstruction of several parts of the body [6,7,8,9,10,11,12,13], with optimal esthetic and functional outcomes. On the other hand, prolonged operative times and the use of general anesthesia, which is frequently necessary for flap reconstruction, may not be feasible for all patients. Moreover, an extensive wound with limited donor tissue, the patient’s wish to not have additional scars at the donor site, systemic diseases of the macro and microcirculation (such as diabetes, vasculitis, and autoimmune diseases) and severe obesity may represent contraindications to flap reconstruction.

Over the past thirty years, many kinds of tissue-engineered skin bio-constructs have been designed to accelerate wound healing by providing a stable extracellular matrix [14]. First developed to treat burned patients [15,16], their use has been expanded over the years to manage different skin and soft tissue defects [17,18,19,20,21,22]. Depending on their structure, similar to autologous skin graft anatomy, they can be categorized as epidermal, dermal or dermo-epidermal.

Dermal substitutes (DSs) are recommended worldwide to treat full-thickness skin defects, as they prepare the wound bed, facilitate cell migration toward neodermis formation and, compared to skin grafting, improve scar appearance [23]. Differences in composition, pore size and degradability are likely to affect the outcomes of wound reconstruction with distinct biopolymers. Few studies in the literature have tried to outline precise indications for different types of products, but definitive guidelines have not been defined yet. For this reason, the surgeon’s choice is often dictated by his own experience.

Integra^®^ (Integra Lifesciences Corp, Plainsboro, NJ, USA) and Pelnac^®^ (Gunze Limited, Kyoto, Japan) have been previously compared in vitro [24,25] and in vivo [26,27]. Wosgrau et al. [26] histologically compared these two DSs in a murine model, finding a reduced granulation tissue and vascular density in the initial phase of the wound healing with Pelnac^®^, while at the 9th day after surgery, both matrices gave similar results in terms of integration, inflammatory response and vascularization. A massive and localized inflammatory reaction associated with the granulation tissue has also been reported with Integra^®^ in a human in vivo study by De Francesco et al. [28]. The same paper [28] showed how both dermal substitutes promote a high secretion of collagen type III fibers with a tissue architecture that is closer to the skin physiology with Pelnac^®^. No differences in cell growth or adhesion were observed, but wound contraction was more marked in the injuries treated with Pelnac^®^, while Integra^®^ seemed to be more efficient in treating deep wounds with bone and tendon exposure [28]. The same conduct in terms of wound contracture has been also found in vitro [24].

While most of the authors have made a histologic and subjective assessment, the aim of our study is to evaluate objectively the skin bio-parametric outcomes of full-thickness defects reconstructed with Integra^®^ and Pelnac^®^ and compare them aiming to detect any existing difference.

## 2. Materials and Methods

This no-profit retrospective observational study was conducted in cooperation between the Plastic and Reconstructive Surgery Unit of AOU Sassari, Italy and the SkinLab of the Department of Biomedical Sciences of the University of Sassari, Italy.

### 2.1. Patients and Methods

**Figure 1:**
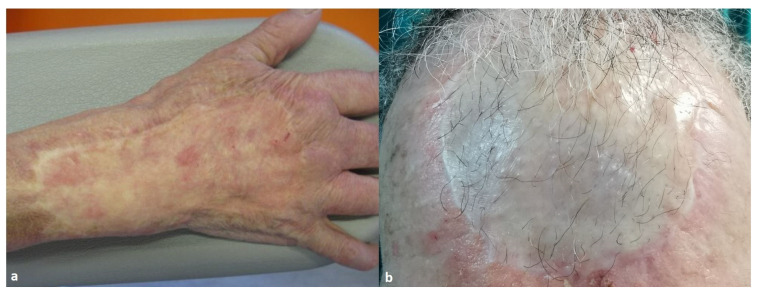
(**a**) Skin reconstructed with Integra^®^ plus FTSG after skin necrosis due to i.v. infusion leak on the dorsum of the hand. (**b**) Skin reconstructed with Pelnac^®^ plus FTSG after tumor resection on the forehead.

We retrospectively analyzed our medical record and selected all the patients who underwent full-thickness wound reconstruction with Integra^®^ or Pelnac^®^, followed by full- or partial-thickness skin graft in a single or double-stage surgery between 2013 and 2020 (Figure 1). The inclusion criteria were patients of either sex, age of >18 years old at the time of the evaluation and follow-up time after the skin grafting > 12 months. The exclusion criteria were patients with vasculopathy, allergic contact dermatitis, atopic dermatitis, ongoing local infection at the time of the evaluation, tumor relapse and skin graft loss. The choice between the two dermal substitutes has been dictated by the surgeon’s own experience and preference.

Integra^®^ is a bilayer system composed of a 2 mm thick inner porous layer made of bovine tendon type I collagen, crossed-linked with shark chondroitin-6-sulfate glycosaminoglycan with a 70 to 200 μm diameter pore size and covered by a temporary epidermal substitute made of silicone [14,28]. Pelnac^®^ is a bilaminar membrane with a 3 mm thick inner atelocollagen sponge derived from pig tendon, with an average pore diameter of 70 to 110 μm and a superficial silicone film layer [14,28]. The latter DS is also available as one layer without the superficial silicone film.

### 2.2. Evaluation Tools

Patients underwent a non-invasive assessment of several skin parameters that were evaluated using the following devices (Courage + Khazaka Electronic GmbH, Köln, Germany):

- Corneometer CM825^®^ to determine the moisture index, which represents the hydration level of the stratum corneum of the skin.
- Tewameter TM300^®^ to assess the transepidermal water loss (TEWL).
- Visioscan^®^ VC98 USB to determine skin texture using the SELS (Surface Evaluation of the Living Skin) parameters.
- Mexameter MX18^®^ to evaluate the melanin and erythema index which provide a reproducible estimate of the content of hemoglobin and melanin, respectively.
- Skin-Colorimeter CL 400^®^ to assess the color of the skin using the CIELAB system.
- Glossymeter GL200^®^ to evaluate the skin gloss.
- Cutometer^®^ dual MPA 580 (probe of 2 mm diameter) to determine the elasticity, using the R-parameters.

The evaluation was made over three different locations on each patient: the grafted skin, the surrounding normal skin (control area) and the skin donor site (donor area).

All the measurements were performed according to the equipment manufacturer’s guidelines and were made under controlled environmental conditions with a mean temperature of 23 °C and a relative humidity of 56%. The measurements to determine the moisture parameter and melanin/erythema index were repeated four times in a row for each evaluated area, and the mean was then calculated. All the other parameters were tested with a single measurement.

### 2.3. Statistical Analysis

The data were entered in Microsoft Excel sheet 2021. The collected data were grouped according to the used dermal substitute. The descriptive statistics (frequency, mean, range and standard deviation) were then calculated for each group and relative to the three different skin areas of evaluation. The normal distribution of quantitative variables was verified using the Shapiro–Wilk test. A paired Student-*t* test was applied to compare skin parameters of the grafted skin to the ones relative to the donor area and the control area. The mean percentage variations between the grafted skin and the donor, or control area of major skin parameters, were then calculated. A value of *p* < 0.05 was considered statistically significant. All statistical analyses were performed using Stata 17.0 software.

## 3. Results

Between 2013 and 2020, forty-nine patients underwent skin reconstruction with Integra^®^ or Pelnac^®^ at our Plastic and Reconstructive Surgery Department. Thirty-four patients were excluded from the study due to inclusion/exclusion criteria, patient loss during follow-up and non-adhesion to the study. Fifteen patients were then included in the study. Demographic and medical/surgical characteristics are listed in Table 1. Eight patients were treated with Integra^®^ and seven with Pelnac^®^ (in one case we used the one-layer product).

**Figure 2:**
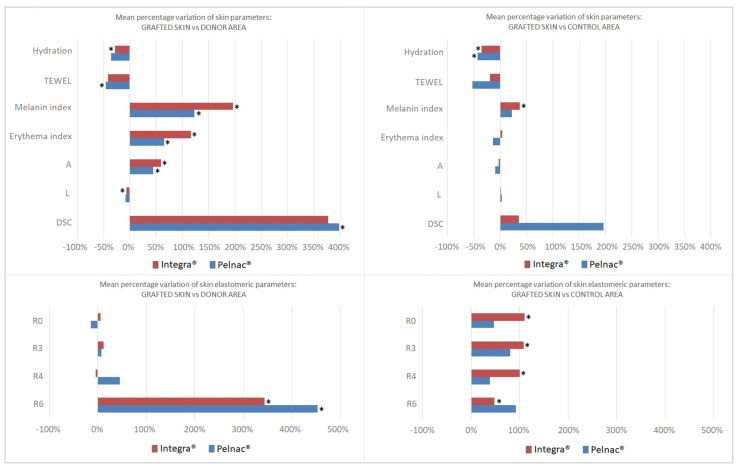
Detected variation of skin parameters between the grafted skin and the donor area on the left and between the grafted skin and the control area on the right are expressed in terms of mean percentage variations. Non-elastic and elastic parameters are listed above and below, respectively. Statistically significant variations are marked with an asterisk.

The reconstructed skin showed changes in different biophysical properties; thirteen out of twenty-five of the evaluated parameters underwent some degree of modifications. Our results are listed in Table 2. The results are also represented in Figure 2 in terms of the mean percentage difference between the reconstructed skin and the donor or control area.

Both dermal substitutes showed a lower degree of hydration when compared to the control area and the bovine-derived substitute showed the same conduct vs. the donor area too. Despite this, the skin barrier function was found to be maintained, as similar or lower values of the TEWL were found in the reconstructed skin.

The superficial skin surface analysis comprised the evaluation of four parameters: skin scaliness (SEsc), skin roughness (SEr), skin wrinkles (SEw) and skin smoothness (SEsm). Both groups of patients showed no statistically significant difference in all evaluated parameters vs. donor and control areas.

In order to quantify skin color, we used the CIELAB system, which is a three-dimensional color space defined by the International Commission on Illumination. This system calculates four values that can be transcribed to dermatological parameters: L* correlates with the brightness of the skin, a* with erythema, b* with skin pigmentation and tanning and ITA with skin types [27]. Both dermal substitutes presented higher levels of melanin concentration with a reduction of the L*-parameter in comparison to the donor area, and the bovine-derived substitute showed a higher pigmentation vs. the control area too. Both DSs also presented a higher level of hemoglobin index and a*-parameter compared to the donor area, while no differences were seen vs. the control area.

The measurement of gloss is based on the reflection of light sent to the skin. The software generates three parameters: direct glossiness, Diffused light and Diffuse Scattering Correction (DSC). The latter one is the value mostly free from the influence of skin color. Both DS skins presented a positive mean percentage variation of DSC in comparison with the control area, which was higher with Pelnac^®^.

The elastic properties of the reconstructed skin were evaluated with the Cutometer^®^. The measuring principle of this device is based on the suction method, where a negative pressure of 450 mbar is applied for 1–3 s through a probe and deforms the skin mechanically. The subsequent skin deformation is displayed as a curve and the software calculates several parameters:

- R0 represents the passive behavior of the skin to external forces which depends on the thickness of the skin and/or its structure, with higher values indicating that the skin is more flexible [29,30]. The skin grafted over Integra^®^ showed higher values vs. the control area, while Pelnac^®^ showed no difference.
- R1 represents the ability of the skin to return to its original state after a deformation. No differences were seen with both DSs.
- R2 represents gross elasticity, with higher values indicating that the skin is more elastic. No differences were seen in both groups.
- R3, R4 and R9 are the parameters most indicative of human skin fatigue [31]. After applying multiple stress deformations, the skin progressively loses the ability to restore its initial position. In our study, the skin reconstructed with the bovine-derived substitute showed higher values of these parameters when compared to the control area, while both DSs showed no difference vs. the donor area.
- R5 represents the net elasticity, with higher values indicating that the skin is more elastic. No differences were seen in both groups.
- R6 represents the portion of visco-elasticity on the elastic part of the curve (the smaller the value the higher the elasticity). Integra^®^ showed higher values of this parameter compared to both the donor and control area, while Pelnac^®^ just vs. the donor site.
- R7 represents the portion of elasticity compared to the complete curve, with higher values indicating that the skin is more elastic. No differences were seen with both DSs.
- R8 represents the ability of the skin to return to a normal state. No differences were seen in both groups.

## 4. Discussion

A considerable amount of artificial dermal substitutes are available nowadays on the market. The aim of these products is to guide the wound healing process in order to recreate an esthetically and physiologically functional skin [15,32]. The key point is to create a non-scar dermal layer that allows neoangiogenesis, extracellular matrix deposition and subsequent engraftment of an autologous skin graft [32,33,34].

Since Pelnac^®^ and Integra^®^ share similar structures and clinical indications, we planned to compare them in a clinical setting, in order to understand the contribution of these products over skin biophysics. Moreover, we tried to compare two of the most known xenogeneic substitutes, aiming to detect any existing difference between them.

As several histologic and subjective assessments of the above-mentioned DSs have already been made by several authors [24,25,26,27,28], we decided to make an objective non-invasive evaluation of several skin bio-physical properties of the skin reconstructed with Pelnac^®^ and Integra^®^. The devices used in our study (see Section 2.2) are used worldwide in dermatology and cosmetology as they have already proved to be reliable tools for an objective functional assessment of the skin [31,35,36,37,38].

In our study, both DSs were effective in restoring skin integrity, and in some cases, the grafted skin was found to be changed in comparison with the donor area, acquiring biophysical features more similar to the recipient skin. In particular, some major changes were found regarding the hydration, color and elastomeric parameters.

Even though the skin barrier function was found to be maintained, both dermal substitutes showed a lower degree of hydration level when compared to the control area. These conflicting results may be explained by a decrease or denervation [39] of sweat glands while the sebaceous gland’s function was maintained. Another possible explanation could be related to a limited subepidermal capillary network or a different [39,40] composition of collagen fibers and GAGs of the inner dermal layer, which retains more water. Our results partially contrast with Nicoletti et al. study’s [27], in which Integra showed a lesser degree of hydration, but a higher water loss than the normal skin. The latter result may be related to the fact that all of their patients were reconstructed with a split-thickness skin graft (STSG), which does not permit the restoration [39] of the adnexal structure of the wound site. On the contrary, most of our patients were reconstructed with a full-thickness skin graft (FTSG), which is able to partially preserve the glands content of the skin, thus, maintaining a functional skin barrier. Moreover, is not clear if the differences detected by Nicoletti [27] are statistically significant, since the *p*-value indicated in the study is about the whole specimen and not about the group of patients reconstructed with the bovine-derived substitute. Park et al. [41] have recently examined two of the most known acellular dermal matrices derived from cadaveric skin, CGDerm^®^ and Alloderm^®^, with a Corneometer^®^ and Tewameter^®^ evaluation. Both dermal substitutes showed a reduction of the hydration level and TEWL parameter, about 15% compared to the surrounding normal skin, displaying similar results as ours.

Hyperpigmentation is one of the most unesthetic attributes that human skin can present and Integra^®^ seems the DS most affected by this variation, while Pelnac^®^ features a better approximation to the surrounding normal skin. This difference may be explained by the higher inflammatory reaction associated with the bovine-derived substitute [26,28], which can lead to a post-inflammatory hyperpigmentation. Contrary to our results, Nicoletti et al. [27] found lower values of melanin content in the skin reconstructed with the bovine-derived substitute, but they did not mention the donor area or the site of injury, thus making a comparison not possible. The difference in the melanin content seen vs. the donor area may be instead explained by the fact that the grafted skin is usually taken from a sun-covered area, while most of the full-thickness defects evaluated in this paper were located over the scalp and forehead. Both DSs also presented a higher erythema index compared to the donor area. This result may be related to the neoangiogenesis phenomenon, which has been described in histological [26,32] and clinical [42] findings.

A superficial skin surface analysis showed no difference in all evaluated parameters, meaning that the skin reconstructed with Integra^®^ and Pelnac^®^ is able to change the texture and conform to the recipient site. Nicoletti et al. [27] also evaluated the skin surface profile in several DSs with a different device and found that Integra^®^ presented the smoothest surface of all specimens and controls.

In cosmetology, skin gloss associated with the sebum film derived from sebaceous glands is deemed unpleasant as it causes a specular surface reflection [43]. Thus, the right balance between hydration (moisture) and sebum (superficial skin lipids) is dermatologically and cosmetically desired [44]. In our case, the reconstructed skin displayed a greater level of shine, probably due to the imbalance between the dehydration of the stratum corneum and the preserved sebum production, as mentioned above.

A major difference between the two DSs was revealed in our study in terms of elasticity. The skin reconstructed with Integra^®^ was found to be more flexible (higher values of the R0 parameter) in comparison to the control area, meaning that the skin preserved the flexibility of the donor skin. Pelnac^®^, instead, acquired the rigidity of the control area, and this may be explained by the higher wound contracture associated with this DS [24,32]. The same result has been found by Nicoletti et al. [27] on the bovine-derived substitute, while Moiemen et al. [39] found a reduction of 61% of the maximal extension of the reconstructed skin vs. the control skin. In the latter study, however, they did not mention the diameter of the probe used for the measurement.

The skin reconstructed with the bovine-derived substitute also showed a major “tiring effect” (higher values of the R3 and R4 parameters) when compared to the control area, while both DSs showed no difference vs. the donor area. The “tiring effect”, which is related to the elastic fibers function, is a characteristic of aged skin [45]. The detected difference between the two evaluated groups may be related to a different distribution of the elastic fibers in the dermal layer, as Moiemen et al. [39] described on Integra^®^, or may be a consequence of the different mean patients’ age of the two evaluated groups (69.8 y. Integra^®^ vs. 60.0 y. Pelnac^®^).

Finally, Integra^®^ also showed higher values of the R6 parameter compared to both the donor and control area, while Pelnac^®^ just vs. the donor site. R6 is a measure of the viscous-elasticity and is the most indicative of water content [31]. Higher values are probably related to changes in the composition of proteoglycans and collagen fibers, which lead to an increase in the dermal water content that decreases the friction between the fibers and facilitates interstitial fluid movements. Our results mean that the skin reconstructed with the bovine-derived substitute should have a higher water content than the control area, which is in contrast with the hydration level found in our specimen. This could be explained by the fact that the Corneometer^®^ probe tests the superficial epidermal layer down to a depth of about 0.1 mm [46], while the Cutometer^®^ reaches deeper layers. In the skin reconstructed with these two DSs, we probably assist in a shift of the water from the superficial layers to the deep ones, probably because of the different composition in collagen fibers and GAGs of the inner layers.

According to our results, in order to obtain a better esthetic outcome, precise post-operative indications should be given to patients who underwent skin reconstruction with Integra^®^ or Pelnac^®^. Patients should be informed of the importance of adequate skin hydration to create a better hydrolipidic film. The use of sunscreen protection and smoothing creams is also important to prevent melanin hyperpigmentation of the grafter skin, particularly with Integra^®^ and in sun-exposed areas.

Due to the differences seen in the elastic properties between the two DSs, Integra^®^ seems to be more advantageous for reconstructing areas that need more skin pliability and flexibility such as joints.

Our study had some limitations. The patient sample size was small and heterogeneous, causing some bias related to different areas of injury, different donor areas for skin grafts, a wide range of patients’ age, different mean follow-up periods and different types of skin grafts (STSG or FTSG). Moreover, the cosmetic habits of the included patients were not analyzed.

## 5. Conclusions

Based on our observational, study both Integra^®^ and Pelnac^®^ are effective means of skin reconstruction and can be used successfully in surgery. Differences have been detected between them in terms of hydration, pigmentation and elasticity, meaning that biomaterials may influence tissue regeneration with different impacts on clinical outcomes.

A larger study is needed to confirm our results and may allow a better understanding of any existing difference between the two examined DSs. This paper could be considered an exploratory work that can settle some important basis for future studies.

## Figures and Tables

**Table 1:** Demographic and medical/surgical characteristics of the patients enrolled in the present study.

	Integra^®^	Pelnac^®^	Total
AGE (mean ± SD in years)	69.8 ± 18.1	60.0 ± 28.3	65.2 ± 23.1
(range)	(30–86)	(18–91)	(18–91)
SEX (male:female)	7:1	6:1	13:2
PRIMARY LESION			
Trauma	0	2	2
Tumour resection (BCC, SCC, Melanoma, Dermatofibrosarcoma)	6	5	11
Scar revision	1	0	1
Infusion leak	1	0	1
SITE OF INJURY			
Scalp	4	3	7
Forehead	2	2	4
Upper limb	1	2	3
Lower extremity	1	0	1
SKIN DONOR SITE (Type of Skin Graft)			
Thigh (STSG)	3	2	5
Groin (FTSG)	1	1	2
Belly (FTSG)	3	1	4
Clavicular region (FTSG)	0	2	2
Arm (FTSG)	1	1	2
FOLLOW-UP PERIOD (mean ± SD in months)	38.3 ± 16.0	16.9 ± 4.7	28.3 ± 16.1
(range)	(12–57)	(12–24)	(12–57)

SD standard deviation; BCC basal cell carcinoma; SCC Squamous cell carcinoma; STSG split-thickness skin graft; FTSG full-thickness skin graft.

**Table 2:** Statistical analysis of evaluated skin bio-parameters.

			Integra^®^			Pelnac^®^		
Device		Parameter	Reconstructed Skin	Donor Area	Control Area	Reconstructed Skin	Donor Area	Control Area
Corneometer	CM825^®^	Moisture index	26.07 ± 11.3	38.51 ± 8.19	46.37 ± 16.7	29.96 ± 20.1	48.41 ± 7.93	58.28 ± 14.1
		*p*-value		***0.0471***	***0.0404***		*0.0762*	***0.0344***
Tewameter	TM300^®^	TEWL	7.037 ± 4.23	15.02 ± 12.3	11.23 ± 4.96	7.171 ± 4.52	13.18 ± 7.73	17.7 ± 14.2
		*p*-value		*0.0987*	*0.1422*		***0.0116***	*0.0503*
Visioscan^®^ VC98		SEsc (Scaliness)	1.503 ± 0.93	0.568 ± 0.34	1.058 ± 0.87	1.02 ± 0.74	0.374 ± 0.47	0.67 ± 0.59
		*p*-value		*0.070*	*0.362*		*0.120*	*0.414*
		SEr (Skin roughness)	3.331 ± 1.39	10.61 ± 19.7	3.123 ± 1.50	6.685 ± 7.71	3.337 ± 1.97	4.014 ± 2.75
		*p*-value		*0.342*	*0.824*		*0.262*	*0.407*
		SEw (Wrinkles)	90.23 ± 29.5	116.4 ± 72.0	148 ± 93.7	181.6 ± 89.7	117.6 ± 42.3	127.1 ± 41.1
		*p*-value		*0.326*	*0.103*		*0.097*	*0.128*
		SEsm (Skin smoothness)	210.2 ± 106	291.5 ± 132	333.4 ± 192	270.3 ± 175	298.0 ± 49.6	289.1 ± 76.9
		*p*-value		*0.203*	*0.088*		*0.675*	*0.805*
Mexameter MX18^®^	Skin-Colorimeter CL 400^®^ & CIELAB system	Melanin index	151.4 ± 41.7	90.28 ± 42.8	116.8 ± 44.9	198.9 ± 132	91.89 ± 48.6	163.7 ± 93.7
		*p*-value		***0.001***	***0.009***		***0.0499***	*0.1548*
		Erythema index	308.9 ± 65.0	184.0 ± 90.0	302.6 ± 75.2	305.9 ± 91.9	184.7 ± 20.7	387.2 ± 136
		*p*-value		***0.002***	*0.741*		***0.010***	*0.168*
		L*	64.81 ± 3.27	69.90 ± 3.89	64.58 ± 3.93	63.10 ± 6.67	68.76 ± 2.90	61.64 ± 6.57
		*p*-value		***0.002***	*0.914*		*0.062*	*0.574*
		a*	8.095 ± 1.85	5.722 ± 1.97	8.967 ± 2.82	8.6 ± 1.43	6.475 ± 2.27	11.01 ± 3.28
		*p*-value		***0.017***	*0.395*		***0.038***	*0.207*
		b*	10.12 ± 2.63	11.42 ± 2.26	11.87 ± 2.86	11.11 ± 3.07	9.792 ± 2.38	11.40 ± 1.22
		*p*-value		*0.232*	*0.059*		*0.396*	*0.868*
		ITA	54.75 ± 11.6	59.5 ± 7.92	50.25 ± 11.1	47.57 ± 21.5	62.14 ± 8.35	42.42 ± 17.9
		*p*-value		*0.204*	*0.367*		*0.174*	*0.513*
Glossymeter GL200^®^		Direct glossiness	8.141 ± 6.69	5.207 ± 1.34	8.195 ± 3.30	11.76 ± 5.10	5.318 ± 0.46	8.264 ± 2.94
		*p*-value		*0.273*	*0.977*		***0.018***	*0.262*
		Diffuse Scattering Correction (DSC)	6.692 ± 5.90	2.932 ± 4.21	5.615 ± 3.39	9.83 ± 5.37	1.962 ± 0.49	6.254 ± 3.36
		*p*-value		*0.178*	*0.555*		***0.010***	*0.270*
		Diffused light	27.2 ± 2.78	37.61 ± 5.12	27.55 ± 3.26	20.7 ± 5.92	33.3 ± 3.50	21.31 ± 6.25
		*p*-value		***0.001***	*0.838*		***0.002***	*0.833*
Cutometer^®^		R0 (Total elongation)	0.085 ± 0.04	0.116 ± 0.05	0.042 ± 0.01	0.092 ± 0.03	0.151 ± 0.05	0.076 ± 0.02
		*p*-value		*0.353*	***0.039***		*0.121*	*0.421*
		R1 (Return to original skin)	0.012 ± 0.00	0.017 ± 0.00	0.009 ± 0.00	0.011 ± 0.00	0.019 ± 0.00	0.016 ± 0.00
		*p*-value		*0.079*	*0.197*		*0.206*	*0.381*
		R2 (Gross elasticity)	0.819 ± 0.10	0.845 ± 0.07	0.798 ± 0.04	0.886 ± 0.08	0.858 ± 0.09	0.785 ± 0.10
		*p*-value		*0.670*	*0.674*		*0.662*	*0.120*
		R3 (Tiring effect)	0.095 ± 0.05	0.123 ± 0.05	0.047 ± 0.01	0.117 ± 0.02	0.154 ± 0.06	0.081 ± 0.03
		*p*-value		*0.436*	***0.040***		*0.335*	*0.119*
		R4 (Tiring effect)	0.028 ± 0.01	0.029 ± 0.00	0.014 ± 0.00	0.024 ± 0.01	0.028 ± 0.00	0.024 ± 0.00
		*p*-value		*0.827*	***0.025***		*0.649*	*0.975*
		R5 (Net elasticity)	0.744 ± 0.25	0.531 ± 0.14	0.661 ± 0.22	0.655 ± 0.29	0.488 ± 0.21	0.530 ± 0.15
		*p*-value		*0.153*	*0.247*		*0.340*	*0.344*
		R6 (Viscoelasticity)	1.022 ± 0.51	0.337 ± 0.13	0.686 ± 0.24	0.630 ± 0.25	0.171 ± 0.09	0.451 ± 0.22
		*p*-value		***0.014***	***0.048***		***0.004***	*0.168*
		R7 (Skin firmness)	0.356 ± 0.06	0.402 ± 0.12	0.383 ± 0.09	0.525 ± 0.18	0.418 ± 0.17	0.367 ± 0.10
		*p*-value		*0.430*	*0.338*		*0.468*	*0.191*
		R8 (Total recovery)	0.072 ± 0.04	0.098 ± 0.05	0.032 ± 0.00	0.080 ± 0.04	0.131 ± 0.06	0.060 ± 0.03
		*p*-value		*0.451*	*0.053*		*0.210*	*0.355*
		R9 (Tiring effect)	0.009 ± 0.01	0.006 ± 0.00	0.005 ± 0.00	0.024 ± 0.02	0.003 ± 0.00	0.004 ± 0.00
		*p*-value		*0.505*	*0.276*		*0.119*	*0.127*

Data are expressed as mean ± standard deviation (SD); statistically significant *p*-values are marked in bold. Italics is used to differentiate the results of the evaluation from *p*-values.

## Data Availability

The data used and/or analyzed during the current study are available from the corresponding author upon reasonable request.
